# Supplementary material for: Polyketide synthase gene clusters in the ericoid mycorrhizal fungus Oidiodendron maius: genome-wide prediction, expression profiling, and functional characterization of knockout mutants
Source: Front Plant Sci. 2026 Sep 16;17:1924430. doi: 10.3389/fpls.2026.1924430 (PMC13623903; doi:10.3389/fpls.2026.1924430)
Supplement: Supplementary file 1 [file SupplementaryFile1.zip › Supplementary Materials/Supplementary Folder 1.zip!/Oidma1_AssemblyScaffolds_Repeatmasked/knownclusterblast/cluster7/jgi.p_Oidma1_153333_mibig_hits.html]

| MIBiG Protein | Description | MIBiG Cluster | MiBiG Product | % ID | % Coverage | BLAST Score | E-value |
| --- | --- | --- | --- | --- | --- | --- | --- |
| EWG54274.1 | hypothetical\_protein | BGC0001190 | Fusaric\_acid\_biosynthetic\_gene\_cluster | 26 | 100.932835821 | 309 | 2.1e-83 |
| BAV19380.1 | NRPS-like\_enzyme | BGC0001390 | LL-Z1272beta\_biosynthetic\_gene\_cluster | 26 | 92.8171641791 | 287 | 4.9e-77 |
| AGN71604.1 | conidial\_yellow\_pigment\_biosynthesis\_polyketide\_synthase | BGC0000027 | Azaphilone\_biosynthetic\_gene\_cluster | 27 | 40.7649253731 | 148 | 3.6e-35 |
| XP\_002384859.1 | NRPS-like\_enzyme,\_putative | BGC0001234\_c2 | 4,4'-piperazine-2,5-diyldimethyl-bis-phenol\_biosynthetic\_gene | 28 | 30.6902985075 | 80 | 1.2e-14 |
